# Supplementary material for: Pilot study of a smartphone-based tinnitus therapy using structured counseling and sound therapy: A multiple-baseline design with ecological momentary assessment
Source: PLOS Digit Health. 2023 Jan 18;2(1):e0000183. doi: 10.1371/journal.pdig.0000183 (PMC9931272; doi:10.1371/journal.pdig.0000183)
Supplement: S1 Protocol — (DOCX) [file pdig.0000183.s002.docx]

S1 Protocol. Study Protocol.

DRKS00030007

Pilot study of a smartphone-based tinnitus therapy using structured counseling and sound therapy: A multiple-baseline design with ecological momentary assessment

**Condition category**

Chronic tinnitus

**Date applied**

16/05/2021

**Prospective/Retrospective**

Retrospectively registered

**Overall trial status**

Completed

**Recruitment status**

No longer recruiting

**Summary**

Background

About 10-20% of the population has chronic tinnitus and 1% suffers very severely. There is no curative therapy, but there are treatment options that can reduce the burden of tinnitus. Due to a lack of therapists, reduced mobility of many patients and structurally weak rural regions, many sufferers do not get access to therapy. Internet-based therapies could close this health care gap.

Objectives and study aims

This pilot study investigates whether the UNITI smartphone app is a feasible and effective treatment option for chronic tinnitus. The UNITI app consists of structured tinnitus counseling, acoustic therapy and an Ecological Momentary Assessment (EMA) module that measures tinnitus symptoms and well-being on a daily basis. A single-case experimental design (SCED) will be used. During a baseline phase, EMA is used to record symptoms on a daily basis before the treatment modules are additionally introduced in an intervention phase (EMA continues). The outcome variables (tinnitus loudness and distress) will be recorded before the baseline phase and after the intervention phase using clinical questionnaires, as well as during both phases using EMA. On the one hand, treatment compliance and app use is being investigated. On the other hand, it will be investigated whether the tinnitus symptoms are reduced by the app intervention. Participants are of age, have chronic tinnitus and at least a mild tinnitus burden.

Study design

Multiple baseline design across 4 groups (from the group of Single-case experimental designs)


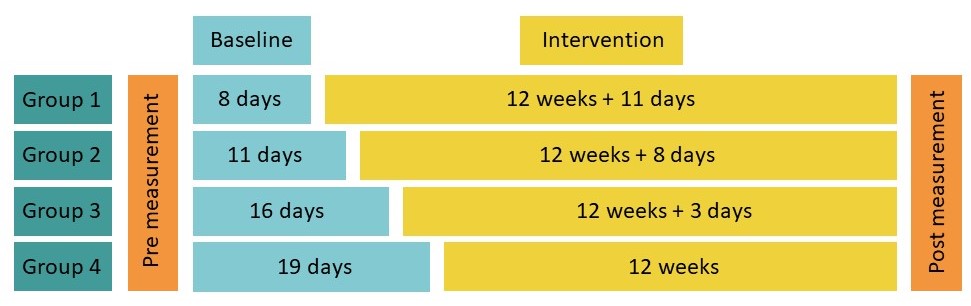


- Two phases: baseline phase (only EMA), intervention phase (EMA, structured counseling, acoustic therapy)
- Staggered introduction of intervention phase to separate treatment effects from random time effects and therefore enhance internal validity
- Intervention phase start is determined randomly (criteria: minimum baseline phase duration of 7 days, distance of 3-5 days to each other)
- Intervention phase: 12 weeks for all groups
- Before baseline phase and after intervention phase: clinical questionnaires

Randomization

Participants are randomly allocated to groups; the order of the groups is randomly determined and the start of the intervention phase is randomly determined (Koehler-Levin method)

Inclusion criteria
1) adult (age ≥ 18 years), 2) chronic tinnitus (duration ≥ 6 months), and 3) THI-score ≥ 18 (indicating at least a mild tinnitus handicap)

Exclusion criteria

1) tinnitus-related therapy within the last 3 months and 2) similar therapy with long term effects e.g., noiser.

Target sample size

minimum 20 participants (to allocate at least 5 participants to each group)

Location

The whole study is conducted online.

Outcome measures

There are two different measurement methods: Clinical questionnaire and Ecological Momentary Assessment

Clinical questionnaire (applied before baseline phase and after intervention phase)

- Tinnitus Handicap Inventory (THI)

Ecological momentary assessment (applied daily during baseline and intervention phase)

- Tinnitus distress measured on a visual analogue scale (0 not burdensome – 100 very burdensome): “How burdensome do you find your tinnitus at the moment?”
- Tinnitus loudness measured on a visual analogue scale (0 inaudible – 100 very loud): “How loud is your tinnitus at the moment?”

Intervention

Structured Counseling: Daily information about tinnitus ranging from basics about hearing and tinnitus to more advanced topics such as therapeutic options. Additionally provided tips on how to better deal with tinnitus and weekly quizzes to test knowledge.

Acoustic therapy: Sound library with natural and digital sounds using different filters i.e. therapeutic approaches. Recommendation to use at least 15 minutes per day.

Statistical analysis

- Descriptive statistics for demographics, tinnitus characteristics and app use
- THI: paired t-test to determine change from pre baseline phase to post intervention phase
- EMA: visualization of tinnitus symptom courses (tinnitus distress and loudness)
- EMA: paired t-test to determine change from baseline phase to end of intervention phase
- EMA: change in relationship between tinnitus distress and loudness over study course
- Comparison of THI with EMA tinnitus distress

Timescale

Recruitment and data collection will probably take place between May and October 2021. Afterwards, results will be analysed and written up for publication.

Ethical approval

The study is approved under the ethic vote 20-1936_2-101 „UNification of treatments and Interventions for TInnitus patients – Randomized Clinical Trial (UNITI-RCT)“ (Amendment No. 2) by the ethics committee of the University of Regensburg, Germany.

Funding

This project receives funding from the European Union's Horizon 2020 Research and Innovation Programme, Grant Agreement Number 848261.

Where is the study run from?
University of Regensburg

Primary contact

Milena Engelke

milena@engelke.me
_____
