# Supplementary material for: Pilot study of a smartphone-based tinnitus therapy using structured counseling and sound therapy: A multiple-baseline design with ecological momentary assessment
Source: PLOS Digit Health. 2023 Jan 18;2(1):e0000183. doi: 10.1371/journal.pdig.0000183 (PMC9931272; doi:10.1371/journal.pdig.0000183)
Supplement: S1 Table — (DOCX) [file pdig.0000183.s003.docx]

**S1 Table. EMA questions**

| Variable | Question | Scale (VAS) |
| --- | --- | --- |
| Tinnitus distress | How burdensome do you find your tinnitus at the moment? | 0 (not burdensome) – 100 (very burdensome) |
| Tinnitus loudness | How loud is your tinnitus at the moment? | 0 (inaudible) – 100 (very loud) |
