# Supplementary material for: Pilot study of a smartphone-based tinnitus therapy using structured counseling and sound therapy: A multiple-baseline design with ecological momentary assessment
Source: PLOS Digit Health. 2023 Jan 18;2(1):e0000183. doi: 10.1371/journal.pdig.0000183 (PMC9931272; doi:10.1371/journal.pdig.0000183)
Supplement: S2 Table — (DOCX) [file pdig.0000183.s004.docx]

**S2 Table. EMA subset demographics**

Participants demographics and tinnitus characteristics of EMA subset

| *Participant demographics* |  |
| --- | --- |
| Participants | *N* = 14 |
| Female, n (%) | 6 (42.9%) |
| Age in years, mean (range) | 52.7 (33 – 68) |
| PHQ-9, mean (SD) | 5.6 (3.7) |
| *Tinnitus characteristics* |  |
| THI, mean (SD) | 38.1 (15.8) |
| Tinnitus duration in months, mean (range) | 184 (20 – 350) |
| GUEF Hyperakusis, mean (SD) | 10.8 (6.7) |
