# Supplementary material for: Pilot study of a smartphone-based tinnitus therapy using structured counseling and sound therapy: A multiple-baseline design with ecological momentary assessment
Source: PLOS Digit Health. 2023 Jan 18;2(1):e0000183. doi: 10.1371/journal.pdig.0000183 (PMC9931272; doi:10.1371/journal.pdig.0000183)
Supplement: Attachment — Submitted filename: Response_to_Reviewers.docx [file pdig.0000183.s005.docx]

Dear editor, dear reviewer,

first of all, we want to start with thanking the editor and the reviewers for the time and effort they put in our manuscript which helped us to improve it substantially. Second, we want to respond to each point raised by the editor and reviewer(s). The revised manuscript with tracked changes and an unmarked version are uploaded as well.

**Journal requirements**

*1. Please amend your detailed Financial Disclosure statement. This is published with the article. It must therefore be completed in full sentences and contain the exact wording you wish to be published.*

This project has received funding from the European Union's Horizon 2020 Research and Innovation Programme, Grant Agreement Number 848261 (ME, JS, CV, SS, MS, SW, RP, BL, WS). TP didn’t receive any funding. The funders had no role in study design, data collection and analysis, decision to publish, or preparation of the manuscript.

*4. In the online submission form, you indicated that your data will be submitted to a repository upon acceptance. We strongly recommend all authors deposit their data before acceptance, as the process can be lengthy and hold up publication timelines. Please note that, though access restrictions are acceptable now, your entire data will need to be made freely accessible if your manuscript is accepted for publication. This policy applies to all data except where public deposition would breach compliance with the protocol approved by your research ethics board. If you are unable to adhere to our open data policy, please kindly revise your statement to explain your reasoning and we will seek the editor's input on an exemption. Please be assured that, once you have provided your new statement, the assessment of your exemption will not hold up the peer review process.*

Thank you for that note. We suspect that this is a misunderstanding. The full data set including the analysis script is already uploaded to a repository (GitHub). With our submission, we declared that “The data that support the findings of this study are openly available on GitHub (https://github.com/MilenaEn/UNITI-SCED-paper). DOI: 10.5281/zenodo.6958301.“ This has not been changed. Please inform us if there are any further ambiguities here.

**Reviewer 1**

Thank you so much for your helpful comments and the time you put into the revision to improve our manuscript, especially the methodology! Let us briefly answer your questions and concerns.

*Did you include patients with normal hearing or hearing loss? Did you include patients with pulsatile tinnitus, somatosensory tinnitus?*

We included adult patients having chronic tinnitus and a certain amount of tinnitus handicap without specifying comorbidities such as hearing loss or tinnitus type as inclusion criteria. We clarified that in the inclusion criteria (L158-160).

*As you mentioned 7 patients didn't received a clinical evaluation, could you add clinical data for the other 11 patients (normal hearing, Meniere's disease, depression/anxiety, ecc.)?*

That is a good point, thank you for drawing attention to that. We added a clinical description of the EMA subset (N = 14) to the Supplemental Material and stated in text, that the subset is comparable with the full sample regarding participants demographics and tinnitus characteristics (L325 – 327).

*How many patients with hyperacusis based on GUEF?*

Thank you for this question. The GUEF does not assess whether an individual has hyperacusis or not, but rather how much the individual is impaired by its hyperacusis. The GUEF score ranges from 0 – 45, with 0 – 9 being a slight impairment, 10 – 15 being moderate impairment, 16 – 23 being severe impairment and 24 – 45 being very severe impairment. The sample has an average score of 10.3 (sd: 6.7) indicating a moderate impairment on the borderline of slight impairment. We added this information in the sample description (L288).

*Did you evaluate the participants during the COVID-19 pandemic?*

We complemented the procedure description with the information that the study was conducted during the COVID-19 pandemic (L165).

**Reviewer 2**

Thank you very much for the time you spent in revising our manuscript! Your detailed review and especially the specific comments must have taken you a lot of work, for us they were very valuable to revise the manuscript.

*1) The multiple-baseline design is unclear to me. I do not understand what the motivation behind this stratification is. What are the hypotheses? Or, is there a future use of this beyond this pilot? L128-135 explain the use of the multiple-baseline design. However, the results do not make use of this stratification and it is not discussed either.*

Thank you for your questions about the design. We assume that by stratification you are talking about the random allocation of the patients to the groups. We hope our following explanation makes it clearer. Single-case designs (which include multiple-baseline designs) live from replication of the effect. A replication is defined as each introduction of the intervention after a baseline phase (because here, a potential effect of the intervention can be detected). However, replicating an effect under the same circumstances (such as time) is critical regarding internal validity. Therefore, the introduction of the intervention is staggered in time to separate intervention effects from random time effects. It is recommended to have at least three replications so we decided to create four groups (= 4 different time points; added in text L132, Krasny-Pacini & Evans, 2018). The random allocation of the groups to the time points and the random allocation of the participants into the groups is again recommended to enhance internal validity (cited in L137). Considering the different groups in the analysis is therefore not necessary, what is significant however, is the difference between the baseline and the intervention phase.

We have used the following articles as a basis for our decisions.

Lobo, M. A., Moeyaert, M., Cunha, A. B., & Babik, I. (2017). Single-case design, analysis, and quality assessment for intervention research. *Journal of neurologic physical therapy: JNPT, 41*(3), 187.

Krasny-Pacini, A., & Evans, J. (2018). Single-case experimental designs to assess intervention effectiveness in rehabilitation: A practical guide. *Annals of physical and rehabilitation medicine, 61*(3), 164-179.

*2) The fundamentals of Ecological momentary assessments involve not only the use of repeated assessments. It is of great importance to gather information about the situation, that is why the word “ecological” is included. In the present study I can see two flaws on that respect. 1) There is no information gathered about the situation where the patients are when they answer the EMA. Are they at home in a quiet place? Are they in public transport? The situation might very much affect the tinnitus perception, at least in terms of loudness. 2) There is a reminder every day at the same time and this might have affect the “moment” when the patients made use of the app, both the EMA and the intervention. Also, it seems that it was limited to only one EMA per day. As it is right now, it looks more like a “daily Momentary assessment” than a formal EMA. The authors can discuss this, the decisions taken and the implications of those decisions.*

Thank you so much for this very valid comment on our study. We agree with the reviewer that Ecological Momentary Assessment is not only about repeated measurement.

1) This is a very valid thought. In fact, the predecessor of this app, TrackYourTinnitus, contains sensors that detect the ambient sound level and for future projects, we should definitely re-expand on that. Unfortunately, in this app, we didn’t assess objective data on the surroundings when patient answered the EMA. However, next to the tinnitus symptoms, the EMA module asks for the subjective condition of the patient, such as stress and emotions, which could allow conclusions to be drawn about the general situation. Evaluating those data together with the related tinnitus symptoms is definitely on our agenda for future projects. We are discussing this in the manuscript (L538-541).

2) Thank you for making that point. We agree with the reviewer that the daily reminder might have affect the moment of answering the EMA module. However, our idea of the EMA module was more that of a daily diary, which was answered by the patients every evening and intended to track the change in tinnitus symptoms influenced by the intervention modules on a daily basis. Our primary research question was the improvement of tinnitus symptoms over the study period, hence, daily EMA was considered sufficient. Furthermore, we tried to keep daily demands on patients as low as possible to maintain long-term compliance. This explanation was incorporated in the method section (L235-238).

Regarding the specific comments, we are only answering those which were not implemented.

*L27: No need of the trial registration in the abstract.*

Thank you for that note. If we are correctly informed, reporting the trial registration details in the abstract is a requirement of the journal for human subject research (<https://journals.plos.org/digitalhealth/s/human-subjects-research>, see Required documentation).

*L37: Do you mean that at the end of the study the loudness might be the same but the distress is lower? (So that is why the correlation is lower).*

Thanks for the enquiry. It is possible that loudness didn’t change during the study period while distress was reduced, our results could indicate that. However, we are careful with any interpretation here, because we have not been able to prove that. Thus, we interpret this finding as a detachment of tinnitus distress from subjective tinnitus loudness.

*L39: I think it should be r= 0.86 because it is the change in EMA*

As you can see in Fig. 6, we defined two different improvement scores in EMA (∆Distress and Distress trend). In the abstract we are referring to association of the THI improvement with both improvement scores. Therefore, it is r = 0.86 and -0.75, respectively.

*L58-59: Already said in the abstract.*

Thank you for making that point. As far as we understood, the Author Summary is intended to be an easier to understand version of the abstract, that is why we repeated ourself here.

*L110: Is there any evaluation of CBT Apps in other field that you could refer to?*

Thank you for considering that. At the moment, one of the authors (WS) is working on a systematic overview of tinnitus apps for a text book where CBT apps are one important part. There are a lot of CBT apps under research, however, we considered that this would expand the introduction. Furthermore, our tinnitus app does not follow a classic CBT approach.

*L142: Staggered introduction seems to be the same as multiple baseline design.*

You have correctly recognized that staggered introduction of the intervention defines a multiple-baseline design. The intention here was to describe the study design in detail.

*L253: Did the daily answers occurred between 19:30-20:00 in the majority of the cases? In that case the EMA might have been influenced by having a reminder every day at the same time.*

Thank you for addressing that. In fact, we see an increased use of the EMA module in the evening hours (17:00 – 21:00). We assume that this is influenced by the notification but also by regular office hours or working schedules. The intention of the EMA module being an end of day diary was therefore fulfilled.

*L264-265: Did you show these results? How did you compare the groups, in different pairs? Perhaps, I got confused and you mean the results in Table 2.*

Thank you for addressing this misunderstanding. We meant the results in Table 2 here, but understood that the wording “group difference” might have caused the confusion and changed it to “sample difference” (L250).

*L362-364 and Table 3 and 4: All the \DeltaDistress, \DeltaTHI etc. When the number is positive, does it mean that the score in the Final visit is larger than in the baseline? Usually, when using ‘change’ (\Delta), it should be End of Intervention phase – Baseline phase. Looking at the footnote of Table 3 I see that here you use a different convention. Therefore, you might want to say that \DeltaXXX is improvement rather than change or just change the signs for consistency. Also, it would be good to remind the readers that a higher number means more handicap, distress and loudness.*

Thank you, that’s a good point. ∆Distress and ∆THI were defined as improvement scores, i.e. positive values indicate an improvement. Therefore, we modified the labeling from change to improvement and mentioned in the method section (L227) as well as in the results (Figure and Table notes) that higher scores indicate more handicap/distress/loudness.

*L407-409. The authors use a mixed linear model as if it was a regression model, to my understanding, these are mathematically similar, but they have different applications. While the first one is usually fitted to the entire dataset and used for exploring effects of factors, regression is used for predictions, so it is important to avoid overfitting and to test the model. Since this is a pilot study, I believe the first approach would be sufficient to explore the effects of the different factors. Alternatively, iterative resampling can be used, and the ranges of the coefficients and p-values used for the analysis.*

Thank you for your feedback. In this paper we used a mixed-effect model due to the hierarchical structure of our data, since we have multiple observations nested within participants. The main difference between a mixed-effect model and a typical OLS (what we believe the reviewer referred to „regression model”), is the ability of mixed-effect models to fit individual intercepts and slopes for each cluster (in our case, each patient was a cluster), and to decompose the variance between the fixed and random effects, which is an integral component of our findings. We agree with the reviewer that analysts should always check for overfitting, especially when dealing with high dimensional data, where p >> n. That was not our case, as we only had a few features included in the model, and we saw no multicollinearity. The model diagnostics showed acceptable fitting and estimates were presented with confidence intervals to help the reader interpret the model coefficients and its ranges.

**Reviewer 3**

Thank you for your time reading and revising our manuscript. We hope we were able to improve it by addressing your concerns.

*1. Introduction is too long to read. Please be short.*

Thank you for indicating that the introduction is too long. We agree with the reviewer that introductions should be kept as short as possible. However, to fully understand the described study, some prior information and previous research citations are required. Trying to balance both requests, we were able to shorten the introduction about half a page.

*2. I cannot understand the meanings of 4 groups. Please explain easily by Figure.*

Thank you for your questions about the 4 groups in our design. Single-case designs (which include multiple-baseline designs) live from replication of the effect. A replication is defined as each introduction of the intervention after a baseline phase (because here, a potential effect of the intervention can be detected). However, replicating an effect under the same circumstances (such as time) is critical regarding internal validity. Therefore, the introduction of the intervention is staggered in time to separate intervention effects from random time effects. It is recommended to have at least three replications so we decided to create four groups (= 4 different time points; added in text L132, Krasny-Pacini & Evans, 2018).

We have used the following articles as a basis for our decisions.

Lobo, M. A., Moeyaert, M., Cunha, A. B., & Babik, I. (2017). Single-case design, analysis, and quality assessment for intervention research. *Journal of neurologic physical therapy: JNPT, 41*(3), 187.

Krasny-Pacini, A., & Evans, J. (2018). Single-case experimental designs to assess intervention effectiveness in rehabilitation: A practical guide. *Annals of physical and rehabilitation medicine, 61*(3), 164-179.
